# The neonatal mortality and its determinants in rural communities of Eastern Uganda

**DOI:** 10.1186/s12978-016-0119-y

**Published:** 2016-02-16

**Authors:** Rornald M. Kananura, Moses Tetui, Aloysius Mutebi, John N. Bua, Peter Waiswa, Suzanne N. Kiwanuka, Elizabeth Ekirapa-Kiracho, Fredrick Makumbi

**Affiliations:** Department of Health Policy Planning and Management, Makerere University School of public Health, Kampala, Uganda; Department of Epidemiology and Biostatistics, Makerere University School of public Health, Kampala, Uganda

**Keywords:** Neonatal mortality, Log-binomial model, Community health workers, Uganda

## Abstract

**Background:**

In Uganda, neonatal mortality rate (NMR) remains high at 27 deaths per 1000 live births. There is paucity of data on factors associated with NMR in rural communities in Uganda. The objective of this study was to determine NMR as well as factors associated with neonatal mortality in the rural communities of three districts from eastern Uganda.

**Methods:**

Data from a baseline survey of a maternal and newborn intervention in the districts of Pallisa, Kibuku and Kamuli, Eastern Uganda was analyzed. A total of 2237 women who had delivered in the last 12 months irrespective of birth outcome were interviewed in the survey. The primary outcome for this paper was neonatal mortality. The risk ratio (RR) was used to determine the factors associated with neonatal mortality using log - binomial model.

**Results:**

The neonatal mortality was found to be 34 per 1000 live births (95 % CI = 27.1–42.8); Kamuli 31.9, Pallisa 36.5 and Kibuku 30.8. Factors associated with increased neonatal deaths were parity of 5+ (adj. RR =2.53, 95 % CI =1.14–5.65) relative to parity of 4 and below, newborn low birth weight (adj. RR = 3.10, 95 % CI = 1.47–6.56) and presence of newborn danger signs (adj. RR = 2.42, 95 % CI = 1.04–5.62). Factors associated with lower risk of neonatal death were, home visits by community health workers’ (CHW) (adj. RR =0.13, 95 % CI = 0.02–0.91), and attendance of at least 4 antenatal visits (adj. RR = 0.65, 95 % CI = 0.43–0.98).

**Conclusions:**

Neonatal mortality in rural communities is higher than the national average. The use of CHW’s to mobilize and sensitize households on appropriate maternal and newborn care practices could play a key role in reducing neonatal mortality.

## Background

To achieve sustainable development goal (SDG) 3 target 3.2, strategies for reducing neonatal deaths must be put in place. Globally 8.2 million children under five die each year, 3.3 million of these deaths occur during the first four weeks of life [1]. In sub-Saharan Africa alone, 1.2 million newborns die every year [2]. This is equivalent to 13,000 deaths per day or almost nine deaths every minute [2]. Neonatal deaths account for an increasing proportion of child deaths, estimated at 41 % by Lawn et al. This unacceptably high rate must be reduced if success towards achieving better child survival is to be reached [2]. In low-income countries nearly half of all mothers and newborns do not receive skilled care during and immediately after birth [1–3]. However two thirds of newborn deaths can be prevented if known effective health measures are provided at birth and during the first week of life [1, 4].

In Uganda, 141, 000 children die before reaching their fifth birthday annually; 26 % of these children die in their first month of life [5]. Between 2000 and 2010, Uganda’s neonatal mortality rate reduced by 2.2 % per year, which is greater than the regional average rate of decline but not good enough to cause significant change in child survival statistics [5]. The major causes of neonatal deaths in Uganda like in other Sub Saharan African countries include; sepsis/pneumonia, tetanus, diarrhea, prematurity, and birth asphyxia [6]. In Uganda, underlying causes of death are related to poor access and low utilization of health services during pregnancy and childbirth [7–10]. As a result more newborn deaths occur at home among the rural poor [9]. To counter these causes of deaths, mothers and newborns need safe and easily accessible care so as to promote the effective management of any arising complications [1].

Timely access to simple interventions such as treating maternal infections during pregnancy, ensuring a clean safe birth, care of the umbilical cord and immediate exclusive breast-feeding could avert most of the newborn preventable deaths [1]. Empowering families and communities to practice safe newborn care practices, to recognize danger signs and early care seeking can help save newborn lives. Community health workers (CHWs) have been used in several low-income settings to sensitize and educate households on a range of health issues and events [11]. In Uganda, the CHW strategy also known as the Village health Team (VHT) strategy has been adopted by the ministry of health to improve newborn care practices within the communities among other functions [10]. This strategy aims at using CHWs to increase health related knowledge and awareness in the communities. Regarding newborn health, CHWs have been used to mobilize pregnant women to attend antenatal, delivery and postnatal care at the nearest health facilities [10]. They also sensitize mothers on a range of care practices and empower them with knowledge to identify danger signs so as to seek appropriate care in time [10].

Recent studies conducted in Uganda have reported stagnated neonatal mortality rates [5]. However, little is known about the neonatal deaths and related risk factors in rural communities. Therefore this study set out to determine the neonatal mortality rate as well as factors associated with neonatal mortality in the rural communities of three districts from eastern Uganda. This is critical, as mortality in rural areas tend to be higher than the national average.

## Methods

### Study design and population

Data for this study was obtained as part of a cross sectional baseline survey conducted for a maternal and newborn intervention hosted at the Makerere University School of public health (MakSPH). The intervention was implemented in Kibuku, Pallisa and Kamuli districts in rural Eastern Uganda. The estimated population in this area is 1,219,172 with an annual population growth rate of 3 %. In July/August 2013, data was collected from the three rural districts using face-to-face interviews from 2237 women who had delivered in the last 12 months irrespective of birth outcomes.

We included women aged 15 to 49 years who had given birth one year prior to the baseline survey. Women whose pregnancies were terminated before 20 weeks and women who were not residents and had not stayed in the community for at least 1 year were excluded from the study. For the purpose of this paper, women with stillbirths were also excluded.

### Sample size and selection of study participants

The study sample size was determined using a two-sided Z-test of the difference between proportions with 80 % statistical power, a 5 % significance level and 1.5 design effect which gave us a sample size of 2293 women. This was based on the intervention’s assumption that, after three years (2013–2015) of implementation, skilled deliveries would increase from 38 to 58 %, 62 to 72 % and 68 to 78 % in the intervention area of Kibuku,^1^ Pallisa and Kamuli districts respectively. To verify if the sample size was good enough to measure neonatal death risk factors, we re-estimated the sample size using openEpi [12] at 95 % confidence interval with an expected neonatal mortality rate of 27/1000 (national average), a precision of 9/1000 (27/1000 ± 9/1000) and a 1.5 design effect which gave us sample size 1867 women. The sample size was therefore sufficient to determine the relationship between home visits by CHWs, health facility delivery, ANC attendance, low birth weight, wealth index, other socio-demographic factors and neonatal mortality. A two-stage cluster sampling technique was applied for each of the study areas. 119 villages were selected using probability proportionate to size sampling techniques, and thereafter all women who had delivered in the last 12 months from the selected villages were listed. All those who were eligible were interviewed. Out of 2293 targeted women 2237 participated in the study survey. During analysis, 184 stillbirths were excluded since our focus was on the factors affecting the neonatal deaths.

### Study variables

The study independent variables include; i) ANC attendance and place where the woman delivered from, ii) socio-demographic factors that included respondent characteristics such as age, education, and parity, iii) newborn characteristics such as presence of danger signs and low birth weight (newborns weighed less than 2.5Kg after birth), iv) newborn mothers who received community health worker’s home visits, and v) wealth index. The wealth index was used to measure equity in health service accessibility. It was developed using principal component analysis. Wealth index grouping was based on household assets, which included ownership of a bicycle, animal, car, motorcycle, phones, chair, table, radio, electricity/solar and the type of the shelter as indicated in the DHS comparative report [13].

### Data collection

A standardized semi structured questionnaire was administered to eligible women after they had given informed consent to participate in the study. Neonatal death was derived from questions on whether the newborn baby died or not within the first 28 days. A team of field staff that included; research assistants, data editors and supervisors were recruited to undertake the study. In order to collect data as completely and efficiently as possible, a 5-day training workshop was conducted. The training covered basic research methodology, study goals, objectives, and use of data collection tools. A team of medical doctors, a statistician and public health specialists conducted the training of the research assistants. On the last day of training, a pre-test of the data collection tools was carried out in Wakiso district that was considered to have the same community characteristics as the three study districts.

### Data management

A data management manual detailing all field data collection procedures, storage and entry was developed for the field staff. During data collection, the data editors checked all the completed questionnaires for errors and missing information. Any error identified was verified and corrected immediately by the research assistants who collected the data. Each supervisor would sample and re-interview 5 respondents each day, in order to check consistency of the information being collected. All data collected were entered using Epinfo 7 software. As one way of checking for consistency of data entry, we double entered 10 % of the questionnaires and compared the two datasets using STATA cf command and no mismatches were identified. Therefore double entry was not done for the rest of the data set. The Epinfo database was backed up and the data was transferred to STATA version 13 (StatCorp LP, College Station, Texas) for analysis.

### Statistical analysis

The analysis was conducted using STATA version 13 (StataCorp 2013). Exploratory data analysis was conducted to obtain descriptive statistics of key variables (frequencies, percentages) prior to bivariate and multivariable analyses. Neonatal mortality was defined as a dichotomous variable. Bivariate analysis was used to determine the association of predictor variables on the newborn’s status after birth. Goodness of fit of the model was assessed using Pregibon’s goodness-of-link test. We also checked for correlation between the independent variables. Risk ratios (RR) with the corresponding 95 % confidence interval were obtained from log-binomial regression. We also examined missing data for all of the study variables. To assess if missing was completely at random or missing at random, we examined the relationship between variables with missing data and other variables.

### Ethical issues

The study obtained ethical approval from the Makerere university school of public health institutional review board (IRB) reference number HDREC 152 and Uganda National Council for Science and Technology (UNCST) reference number HS 1399. Permission was also sought from the district health offices to conduct the study in the 3 participating districts. Community leaders in the participating districts were also informed about the study during mapping. Each individual study participant signed translated consent form, upon consenting to take part in the study. For women aged 15–17 years, further consent was sought from their guardians or parents. The inclusion of women aged 15–18 in this study was part of the study protocol approved by MakSph IRB and UNCST who were considered emancipated minors.

## Results

### Characteristics of study participants

**Fig. 1:**
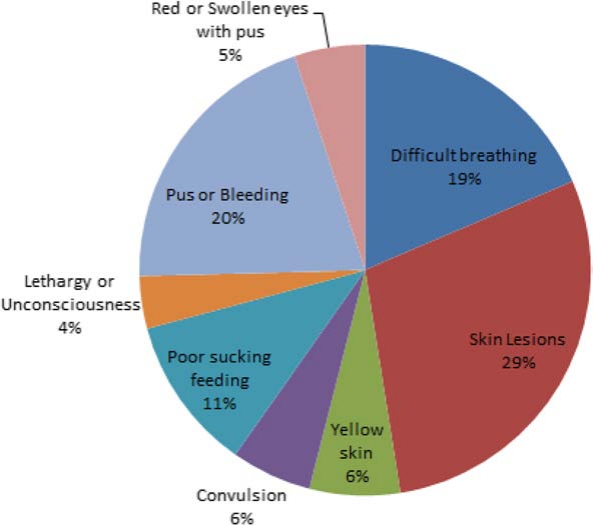
Newborn danger signs experienced

A total of 2237 women who had delivered in the last 12 months were interviewed. 7.5 % of the women were less than 20 years of age and the majority (59.1 %) were in the 20–29 years age group, the 35–49 years age group represented the least percentage (14 %) respectively. The mean age at the time of the last delivery was 27 years. Of these, 91.8 % (2053) and 8.2 % (184) had live births and stillbirths respectively. 23 % of the women had primary education and a few (8 %) had attained secondary level education. Most of the women (82 %) were peasant farmers and few (8 %) had paid occupation. About 45 % of the respondents had carried at least 5 pregnancies at the time of the survey. Majority (94 %) of the respondents had attended antenatal care (ANC) at least once but only 43.6 % had attended at least 4 ANC visits for the most recent pregnancy. The most reported experienced newborn danger signs were (Fig. 1); Skin lesions (29 %), pus or bleeding (20 %) and difficult breathing (19 %).

### Neonatal mortality rate and risk factors at bivariate level

The overall neonatal mortality rate in the three districts was 34/1000 live births (95 % CI = 27.1–42.8). Comparing the three district, NMR was 31.9/1000 live births (95 % CI = 21.8–46.4) in Kamuli, 36.5/1000 live births (95 % CI = 26.6–49.7) in Pallisa and 30.8/1000 live births (95 % CI = 19.8–42.2) in Kibuku. Majority of the deaths occurred within 6 h of delivery (44.7 %), while 30.9 % occurred 7 h to 6 days later, and 24.5 % within 7 to 28 days. Table 1 presents bivariate analysis outputs. Newborns whose mothers were of the age group of 35–39 years were more likely to die relative to those whose mothers belonged to the 25–29 years age group. Newborns whose mothers belonged to 15–19 years age group were less likely to die relative to those who belonged to 25–29 years age group. The risk of newborn death increased significantly with mothers’ parity of 5 and above compared to those whose mothers’ parity was 4 and below (Table 1).

**Table 1:** Socio-demographic, health and physical factors of respondents

Variables	Died/live birth total	Newborn death per 1000 live births	Un-adjusted RR (95 % CI)	*P*-value
Overall	70/2053	34.10		
Age at the interview				
15–19	1/317	3.20	0.10 (0.01–0.77)	
20–24	17/ 604	28.10	0.91 (0.45–1.81)	
25–29	15/ 488	30.70	1.00	<0.001
30–34	16/ 357	44.80	1.46 (0.73–2.90)	
35–39	13/ 200	65.00	2.11 (1.02–4.35)	
40 and above	8/ 87	92.00	2.99 (1.31–6.83)	
Marital Status				
Single	1/99	10.10	1.00	0.27
Married	68/ 1889	36.00	3.60 (0.51–25.67)	
Widowed/Divorced/separated	1/65	15.40	1.54 (0.98–24.16)	
Education level				
Post primary	3/ 166	18.10	1.00	0.21
None	54/ 1393	38.80	2.15 (0.68–6.78)	
Primary	13/ 494	26.30	1.46 (0.42–5.05)	
Occupation				
Income earner	6/195	30.80	1.00	0.67
Peasant farmer	60/ 1686	35.60	0.63 (0.18–2.23)	
House Wife	4/ 172	23.30	0.96 (0.42–2.19)	
Religion				
Protestant	28/ 918	30.50	1.00	0.80
Catholic	21/ 500	42.00	1.38 (0.79–2.39)	
Muslims	9/ 311	28.90	0.95 (0.45–1.89)	
Pentecostal	11/ 296	37.20	1.21 (0.61–2.41)	
Others	1/28	35.7	1.17 (0.16–8.29)	
Quintile				
1 (Poorest household)	19/ 411	46.20	1.00	0.60
2	11/420	26.20	0. 57 (0. 27–1.18)	
3	12/ 387	31.00	0. 67 (0.33–1.36)	
4	14/ 407	34.40	0. 74 (0.38–1.46)	
5	14/ 428	32.70	0.64 (0.32–1.32)	
Parity				
Parity less than 5	22/ 1178	18.70	1.00	
Para 5 and above	48/ 875	54.90	2.94 (1.79–4.83)	<0.001
Number of ANC visits				
ANC <4 times	52/1169	44.50	1.00	
ANC 4 times and above	18/884	20.40	0.45 (0.26–0.78)	0.004
Time of ANC access				
< =3 month	10/517	19.30	0.50 (0.26–0.96)	0.037
4 and above	60/ 1536	39.10	1.00	
Place of delivery				
Health facility	47/1353	34.70	1.00	
Community	23/700	32.90	0.95 (0.58–1.54)	0.824
Baby experienced danger signs				
No	11/572	19.30	1.00	
Yes	59/ 1481	39.80	2.07 (1.10–3.91)	0.025
Newborn with Low birth weight				
Yes	7/ 109	64.20	2.18 (1.01–4.72)	0.048
No	44/1493	29.50	1.00	
Missing	19/ 451	42.10		
Received CHWs home visits				
Never received a CHWs visit	68/ 1810	38.10	1.00	
Received CHWs visit	2/ 243	12.30	0.23 (0.05–0.89)	0.033

*CI* confidence interval, *RR* Risk Ratio

Mothers who attended ANC at least four times, and went for ANC within the first trimester were less likely to lose a newborn compared to those who didn’t (Table 1). The risk of newborn death was two times higher among newborns who experienced danger signs and had low birth weight compared to those who didn’t (Table 1). Community health workers visits to mothers indicated to have significantly reduced the risk of newborn death by 70 % relative to newborns whose mothers never received a visit from community health worker. There was no difference in neonatal deaths among mothers whose delivery was assisted by skilled personnel compared to those whose delivery was assisted by unskilled personnel.

### Factors affecting neonatal mortality at multivariable analysis

Table 2 presents multivariable analysis outputs. The following risk factors were significantly associated with neonatal death at multivariable analysis. Newborns with low birth weight were 3 times more likely to die within 28 days (adj. RR = 3.10, 95 % CI = 1.47–6.56). Additionally, newborns who experienced danger signs after delivery were two times more likely to die compared to those who never experienced any danger sign (adj. RR = 2.42, 95 % CI = 1.04–562). Women who attended ANC at least four times were less likely to lose their newborn (adj. RR = 0.65, 95 % CI = 0.43–0.98). Furthermore, women who were of parity 5 and above were 3 times more likely to lose their newborns within 28 days compared to those who were of parity 4 and below (adj. RR = 2.53, 95 % CI = 1.14–5.65). Also, women who were visited by community health workers were 87 % less likely to lose their newborns compared to those who were not visited by the community health worker (adj. RR = 0.13, 95 % CI = 0.02–0.91).

**Table 2:** Multivariable analysis for predictors of newborn mortality

Variables	Un-adjusted RR (95 % CI)	Adjusted RR (95 % CI)	*P*-value
Age group			
15–19	0.10 (0.01–0.77)	0.15 (.02–1.20)	0.074
20–24	0.91 (0.45–1.81)	0.73 (0.30–1.81)	0.502
25–29	1.00	1.00	
30–34	1.46 (0.73–2.90)	1.04 (0.50–2.17)	0.914
35–39	2.11 (1.02–4.35)	0.67 (0.26–1.77)	0.425
40 and above	2.99 (1.31–6.83)	1.89 (0.76–4.74)	0.173
Parity			
Para less than 5	1.00	1.00	
Para 5 and above	2.94 (1.79–4.83)	2.53 (1.14–5.65)	0.023
Received CHWs home visits			
Never received a CHWs visit	1.00	1.00	
Received CHWs visit	0.23 (0.05–0.89)	0.13 (0.02–0.91)	0.040
Newborn experienced danger signs			
No	1.00	1.00	
Yes	2.07 (1.10–3.91)	2.42 (1.04–5.62)	0.040
Time accessed ANC			
< =3 month	0.50 (0.26–0.96)	0.76 (0.38–1.55)	0.454
4 and above	1.00	1.00	
Newborn with Low birth weight			
Yes	2.18 (1.01–4.72)	3.10 (1.47–6.56)	0.003
No	1.00	1.00	
ANC visits number			
ANC <4 times	1.00	1.00	
ANC 4 times and above	0.45 (0.26–0.78)	0.65 (0.43–0.98)	0.040

## Discussion

In this study, NMR in the three rural districts of Eastern Uganda was found to be higher (NMR = 34 per 1000 live births) than the national average that is estimated at 27/1000 live births [14]. The higher rate could be explained by the fact that this study was done in a predominantly rural area while the national average includes both rural and urban areas. This therefore points to the fact that mortality among newborns could be higher in rural areas compared to the urban areas. The majority (75 %) of the newborn deaths were found to have occurred in the first week of life, 44 % of which occurred on the day of delivery within 6 h. This is consistent with other studies carried out in similar settings in Africa [10, 15]. The first day and first week of life is therefore a critical period that needs to be targeted with effective interventions at both facility and community level [3].

Attendance of ANC at least four times was found to be independently associated with a lower risk of newborn death. This is because additional antenatal visits may serve to reinforce maternal education and compliance, and provide an opportunity for screening and treatment danger signs and infections respectively [16]. Furthermore, health workers can teach mothers to recognize danger signs during pregnancy, labor, and delivery and encourage them to plan clean and safe deliveries—preferably with trained assistance [17, 18]. This is in keeping with existing evidence which indicates that attending ANC at least four times may lead to timely diagnosis and treatment of maternal infections and other health problems during pregnancy which can lead to significant improvement in fetal and neonatal outcomes, as well as prevention of maternal mortality and morbidity [17].

This study revealed that women who were visited by community health workers’ (CHWs) were less likely to lose their newborns. Availing information to women during pregnancy allows them to identify risky pregnancies and therefore increase chances of timely seeking appropriate care. This increases opportunities for early management of maternal and newborn related complications hence contributing to the reduced risk of neonatal mortality [10, 11, 19]. Similarly, promoting strategies that support groups to provide home visitation along with community mobilization, have a significant impact on reducing neonatal mortality [20].

Low birth weight (less than 2.5Kg), which is mostly an indicator for prematurity, was associated with higher risk for neonatal death. Low birth weight (LBW) pre disposes newborns to increased risk of infections, low blood sugar and low body temperatures, which increase the risk of death compared to normal newborns [21]. A need to strengthen the use of kangroo mother care (KMC) practices in rural health facilities as a low cost intervention in resource limited settings for taking care of LBW babies is therefore apparent [22–24]. In this study, a considerate percentage of newborns (22 %) could not have their weight measured, yet this was vital in identifying neonates that deserved special care.

This study also showed that newborns who experienced danger signs were more likely to die within 28 days. The most reported danger signs were (Fig. 1); Skin lesions, pus or bleeding and difficult breathing. These have been reported as preventable conditions if care is sought from skilled health providers. However, mothers’ responses to such danger signs has often been shown to depend on their judgment of the severity of the signs [9]. They commonly start with locally available herbs and if these fail to help, they resort to seeking care from traditional birth attendants or other traditional healers [9] before they finally seek care in the main stream health facilities. The knowledge of newborn danger signs is therefore essential for community members, so as to facilitate appropriate care seeking behavior [25, 26].

Furthermore, mothers with parity of 5 and above had a higher risk of neonatal death compared to those with parity of 4 and less. This was consistent with other studies which indicate that frequent births as well as high parity predispose both the mothers and newborns to higher risks of unfavorable health outcomes [27, 28]. Family planning interventions in especially rural areas could therefore contribute to the reduction such high risk pregnancies [29].

Finally, the risk of neonatal death was not significantly different between women who delivered from health facilities and those who delivered from home. This differs from a study done in Rufigi Tanzania that indicated place of delivery as being associated with reduced risk of newborn deaths [30]. However, it is consistent with two other studies that were also done in Tanzania [31, 32]. These findings suggest that the quality of newborn care in the rural facilities was very poor hence there was no difference in neonatal deaths for babies born at health facilities when compared to those born at home. The rural health facilities are often understaffed [33] and lack essential or basic lifesaving equipment such as resuscitation kits for newborns [32]. Furthermore, delivering at a health facility is sometimes not aided by qualified and skilled personnel, therefore the care given is inadequate [1, 4, 34, 35]. Studies done in low-income countries like Pakistan have also indicated that sub-standard care, inadequate training, low staff competence and a lack of resources, including equipment and medication as contributing factors to neonatal death [2, 14, 33]. This confirms the findings elsewhere that assert that without improvement in the quality of care provided, increased health-care coverage is unlikely to substantially improve neonatal outcomes [30]. Attention to improving quality of care in mainstream health facilities cannot therefore be over emphasized.

## Conclusions

Neonatal mortality in these rural communities was higher than the national average. The risk factors that were significantly associated with neonatal death were: parity of 5 and above, home visits by community health workers, low birth weight, Attendance of ANC at least four times and presence of newborn danger signs. It is therefore recommended that stakeholders should rally together to promote the use of CHW’s who are useful for community mobilization and sensitization leading to increased knowledge on newborn danger signs. This in turn facilitates appropriate care seeking behavior for such newborns as well as attendance of ANC for at least four times as recommended by WHO. In addition stakeholders should promote the use of family planning so as to reduce the high parity. Lastly, understanding the factors associated with low birth weight so that they can be targeted more effectively is yet another critical element to be considered by the different stakeholders.

### Methodological implications

The sample size determination depended on the proportion of facility delivery rather than considering the neonatal death proportion. However, during listing we reached all households in the selected villages and all women who had delivered in the last 12 months were listed and thereafter interviewed. This indicates that the sample size was large enough to estimate neonatal mortality rate. These results are generalizable to eastern Uganda rural communities’ and other low-income countries with a similar context. Additionally, recall bias might have affected the study results but we tried to minimize this by including only women who had delivered in the last 12 months.

## Abbreviations

ANC: antenatal care
CHWs: community health workers
CI: confidence interval
IRB: Institutional Review Board (IRB)
MakSPH: Makerere University School of Public Health
NMR: neonatal mortality rate
RR: risk ratio
SDG: sustainable development goals
TBAs: traditional birth attendants
UNCST: Uganda National Council for Science and Technology
WHO: World Health Organization

## Footnotes

1 The entire Kibuku district was taken as an intervention area
